# Lethal Influenza Virus Infection in Macaques Is Associated with Early Dysregulation of Inflammatory Related Genes

**DOI:** 10.1371/journal.ppat.1000604

**Published:** 2009-10-02

**Authors:** Cristian Cillóniz, Kyoko Shinya, Xinxia Peng, Marcus J. Korth, Sean C. Proll, Lauri D. Aicher, Victoria S. Carter, Jean H. Chang, Darwyn Kobasa, Friedericke Feldmann, James E. Strong, Heinz Feldmann, Yoshihiro Kawaoka, Michael G. Katze

**Affiliations:** 1 Department of Microbiology, School of Medicine, University of Washington, Seattle, Washington, United States of America; 2 Division of Zoonosis, Department of Microbiology and infectious Diseases, Graduate School of Medicine, Kobe University, Kobe, Japan; 3 Special Pathogens Program, National Microbiology Laboratory, Public Health Agency of Canada; 4 Department of Medical Microbiology, University of Manitoba, Winnipeg, Manitoba, Canada, Winnipeg, Manitoba, Canada; 5 Department of Pathobiological Sciences, School of Veterinary Medicine, University of Wisconsin-Madison, Madison, Wisconsin, United States of America; 6 Department of Special Pathogens, International Research Center for Infectious Diseases, Institute of Medical Science, University of Tokyo, Tokyo, Japan; 7 Division of Virology, Department of Microbiology and Immunology, Institute of Medical Science, University of Tokyo, Tokyo, Japan; 8 ERATO Infection-Induced Host Responses Project, Japan Science and Technology Agency, Saitama, Japan; 9 Washington National Primate Research Center, University of Washington, Seattle, Washington, United States of America; Washington University School of Medicine, United States of America

## Abstract

The enormous toll on human life during the 1918–1919 Spanish influenza pandemic is a constant reminder of the potential lethality of influenza viruses. With the declaration by the World Health Organization of a new H1N1 influenza virus pandemic, and with continued human cases of highly pathogenic H5N1 avian influenza virus infection, a better understanding of the host response to highly pathogenic influenza viruses is essential. To this end, we compared pathology and global gene expression profiles in bronchial tissue from macaques infected with either the reconstructed 1918 pandemic virus or the highly pathogenic avian H5N1 virus A/Vietnam/1203/04. Severe pathology was observed in respiratory tissues from 1918 virus-infected animals as early as 12 hours after infection, and pathology steadily increased at later time points. Although tissues from animals infected with A/Vietnam/1203/04 also showed clear signs of pathology early on, less pathology was observed at later time points, and there was evidence of tissue repair. Global transcriptional profiles revealed that specific groups of genes associated with inflammation and cell death were up-regulated in bronchial tissues from animals infected with the 1918 virus but down-regulated in animals infected with A/Vietnam/1203/04. Importantly, the 1918 virus up-regulated key components of the inflammasome, NLRP3 and IL-1β, whereas these genes were down-regulated by A/Vietnam/1203/04 early after infection. TUNEL assays revealed that both viruses elicited an apoptotic response in lungs and bronchi, although the response occurred earlier during 1918 virus infection. Our findings suggest that the severity of disease in 1918 virus-infected macaques is a consequence of the early up-regulation of cell death and inflammatory related genes, in which additive or synergistic effects likely dictate the severity of tissue damage.

## Author Summary

The world is currently experiencing a pandemic caused by a new type of H1N1 influenza virus that originated in swine. Although it is too early to tell how virulent this new virus may be, some influenza viruses can cause severe disease. The 1918 pandemic, also caused by an H1N1 virus, resulted in over 50 million deaths worldwide. Highly pathogenic avian H5N1 influenza viruses are circulating in several parts of the world, and although human infection has been rare, the virus often causes a lethal illness. To determine whether highly pathogenic influenza viruses cause disease by similar means, we used a macaque infection model to study the host response to the reconstructed 1918 virus and an avian H5N1 isolate known as VN/1203. We found that although both viruses caused severe disease, infection with the 1918 virus was more likely to result in death, whereas animals infected with VN/1203 recovered from infection. In animals infected with the 1918 virus, there was increased expression of genes associated with inflammation, and these genes were turned on as early as 12 hours after infection. Drugs that limit the early inflammatory response may therefore be of benefit in treating infections caused by highly pathogenic influenza viruses.

## Introduction

Influenza virus causes over 36,000 deaths [1] and 1.68 million hospitalizations in the United States every year [2]. However, the potential of this virus to disrupt society is best evidenced by the 1918–1919 influenza pandemic, which resulted in over 50 million deaths worldwide. The circulation of highly pathogenic avian H5N1 strains in Asia, and reports of their sporadic human-to-human transmission [3],[4], has raised concern over the potential for a deadly new influenza pandemic. Presently, a novel swine-origin influenza A (H1N1) virus (S-OIV) is spreading rapidly among humans, presenting the greatest threat since the emergence of influenza A (H3N2) virus in 1968 [5]. Although the World Health Organization has recently declared the first pandemic of the new millennium caused by this new S-OIV, initial analyses suggest that the clinical severity associated with the H1N1 virus is less than that seen in 1918 [6]. Nevertheless, preparedness for the current as well as future pandemics will require a better understanding of the host response to influenza virus infection, including mechanisms of viral pathogenesis and interactions with the host machinery, which will facilitate efforts to develop safe and effective therapeutics and vaccines.

Traditionally, studies of influenza virus pathogenesis compare viruses of high and low pathogenicity [7],[8]; nonetheless, comparisons of highly pathogenic strains are necessary to determine commonalities and differences in the host response and to develop effective countermeasures. Results from independent studies using macaque infection models suggest there are differences in lethality and disease progression between the 1918 and H5N1 viruses [8],[9]. In particular, infection with the 1918 virus leads to severe disease and death, whereas animals infected with VN/1203/04, an H5N1 avian virus, typically recover from infection. We have previously shown that an atypical host innate immune response may contribute to the severity of disease and fatal outcome in 1918-virus infected macaques [8]. Additional studies have suggested that early events during infection may be critical for disease outcome [10]. Microarray studies of H1N1 (A/Texas/36/91) influenza virus-infected pigtailed macaques (*Macaca nemestrina*) [11],[12] indicate that activation of inflammatory cells and apoptotic pathways correlates with tissue damage during infection.

In the present study, we examined two highly pathogenic influenza viruses, the fully reconstructed human H1N1 “Spanish flu” 1918 virus (1918) [8] and the highly pathogenic avian H5N1 A/Vietnam/1203/04 virus (VN/1203) in macaques (*Macaca fascicularis*), an established model for influenza virus infection [13],[14]. The Influenza A genome consists of eight gene segments designated PB2, PB1, PA, HA, NP, NA, M, and NS. The identities of these genes between 1918 and VN/1203 virus are 84, 84, 86, 63, 86, 82, 90, and 89%, respectively. We found specific differences in gene expression in the bronchus of animals infected with the 1918 virus compared with those infected with VN/1203, with the largest differences in expression occurring at 24 h after infection. Biochemical assays showed that both viruses elicited an apoptotic response in lungs and bronchi, although the response occurred earlier during 1918 virus infection. These results suggest that the differential regulation of genes associated with specific biological functions, including cell death and inflammation, are associated with increased severity of disease caused by the 1918 virus.

## Materials and Methods

### Ethics statement

All animal experiments were performed under an approved animal-use document and according to the guidelines of the Canadian Council on Animal Care.

### Viruses

The highly pathogenic H5N1 virus used in this study (A/Vietnam/1203/04) was kindly provided by the Centers for Disease Control and Prevention. Genes of the 1918 virus (GenBank DQ208309, DQ208310, DQ208311, AF117241, AY744935, AF250356, AY130766, AF233238) were constructed with the 5′ and 3′ non-coding sequences of A/WSN/33 (H1N1) and cloned into plasmid vector pPolI, as previously described [15]. The 1918 virus was generated by reverse genetics [16], and titered stocks were prepared [15], as previously described. Forty-eight hours post-transfection, viruses were harvested and used to inoculate MDCK cells for the production of stock viruses. Eight genes of each transfectant virus were partially sequenced to confirm the origin of the gene.

### Macaque experiments

The macaques (*Macaca fascicularis*) used in this study were inoculated by intratracheal (4 ml), intranasal (0.5 ml/nostril), intraocular (0.5 ml/eye) and oral (1 ml) routes with a suspension containing 10^6^ plaque-forming units (PFU) per ml for a total infectious dose by all routes of 7×10^6^ PFU. Six animals received the 1918 virus and were euthanized at 12, 24, or 48 h post infection (2 animals per time point). Eleven animals received the A/Vietnam/1203/04 virus and were euthanized at 12, 24, or 48 h or at 3 or 6 days post-infection (2 animals per time point). A single animal infected with A/Vietnam/1203/04 was euthanized at day 16. Viral titers in bronchus were quantified by standard plaque assay in MDCK cells. Tissue samples were placed in RNA Later (Ambion) for subsequent RNA extraction (Qiagen RNA Later kit).

### Histopathology

The tissues were fixed in 10% neutralized phosphate-buffered formalin. Fixed tissues were dehydrated, embedded in paraffin, and cut into 5-µm-thick sections and then stained with standard haematoxylin and eosin. For viral antigen detection, sections were processed for immunostaining by the two-step dextran polymer method (DAKO), with a rabbit polyclonal antibody to A/WSN/33 (H1N1) used as the primary antibody.

### Virus titrations of nonhuman primate tissues and swabs

Tissue homogenates were prepared in minimal essential medium supplemented with 0.1% bovine serum albumin and antibiotics (MEM/BSA) by using a bead mill homogenizer (Qiagen Tissuelyser) at 30 Hz for 5 min. Tissue debris was pelleted by centrifugation (2000×g, 5 min) and virus titers were determined in 10-fold serial dilutions by standard plaque assay on Madin Darby canine kidney (MDCK) cells in the presence of 1 µg/ml tosyl phenylalanyl chloromethyl ketone (TPCK)-treated trypsin, in duplicate for each dilution. Virus in swabs was similarly determined in undiluted and serial 10-fold dilutions of the swab suspension medium.

### Microarray analysis and bioinformatics

Separate microarrays were run for each experimental sample (one sample per animal and two animals per time point). Equal masses of total RNA isolated from bronchi collected from infected macaques were amplified with a Low RNA Input Linear Amplification Kit (Agilent Technology) according to the manufacturer's instructions. Global gene expression in infected bronchi was compared to pooled RNA prepared from equal masses of total RNA from bronchi tissue of six uninfected macaques. Probe labeling and microarray slide hybridization were performed as described elsewhere [17] with custom rhesus macaque (*Macaca mulatta*) oligonucleotide microarrays containing 22,000 rhesus probes corresponding to 18,000 unique rhesus genes (designed in collaboration with Agilent Technologies). Raw microarray image files were processed using Feature Extraction 8.1 software (Agilent Technologies) and entered into a custom-designed relational database (Expression Array Manager). We processed and analyzed the expression data using the limma package for the R programming environment [18]. Background correction was performed by using limma's “normexp” method, which ensures that there were no missing or negative corrected intensities. An offset of 50 was used for both channels to damp down the variability of the log-ratios for low-intensity spots. The resulting log-ratios were normalized by using ‘loess” methods. Between –array normalization was achieved by using limma's “Aquantile” method, which does quantile normalization of mean probe intensity values. The normalized expression data were analyzed together by using linear model methods. A batch factor was included in the linear model to remove potential batch effects. Differential expression was assessed by using moderated t-statistics. Primary data is available at http://viromics.washington.edu in accordance with proposed MIAME standards.

Functional and network analysis of statistically significant gene expression changes was performed with Ingenuity Pathways Analysis 7.1 (Ingenuity Systems). Genes from the data set that met the twofold (*P*<0.01) change cutoff and were associated with biological functions in the Ingenuity Pathways Knowledge Base were considered for analysis. For all analyses, Fisher's exact test was used to calculate a *P*-value determining the probability that each biological function assigned to that data set was due to chance alone. In the functional network shown in this paper, genes are represented as nodes, and the biological relationship between two nodes is represented as an edge (line). All edges are supported by at least one published reference or from canonical information stored in the Ingenuity Pathways Knowledge Base. For these analyses, Fisher's exact test was used to calculate a *P*-value determining the probability that each biological function and/or disease assigned to that data set was due to chance alone.

### Quantitative real-time RT-PCR

RT-PCR was performed to validate cellular gene expression changes as detected with microarrays. The QuantiTect reverse transcription kit (Qiagen Inc., Valencia, CA) was used to generate cDNA. qRT-PCR was run on the ABI 7500 PCR system, using TaqMan chemistry (Applied Biosystems, Foster City, CA). Gene expression assays specific to Rhesus cellular genes were purchased from Applied Biosystems. Differences in gene expression are represented as Log_10_RQ relative to a calibrator and normalized to a reference, using the 2^−ΔΔCt^ method [19].

### TUNEL assay

Lung and bronchus tissues were fixed in 10% neutralized phosphate-buffered formalin. Fixed tissues were dehydrated, embedded in paraffin, cut into 5 µm-thick sections, and then processed for TUNEL assay (X2044K2. ApoMark™ DNA Fragmentation Detection Kit. The Exalpha Biologicals, Inc., Maymard, MA). Briefly, the sections were rehydrated and permeabilized using Proteinase K solution. Endogenous peroxidases were inactivated with 3% hydrogen peroxide in methanol. After terminal deoxynucleotidyl transferase (TdT) reaction, biotinylated dUTP were visualized using HRP-conjugated streptavidin and 3.3′-Diamino benzidine (DAB). The sections were counterstained with methyl green solution.

### Biocontainment

All *in vitro* and *in vivo* procedures with the 1918 and VN/1203 influenza viruses were performed in the biosafety level 4 facility of the National Microbiology Laboratory of the Public Health Agency, Canada. Prior to removal from BSL4, all specimens were inactivated using Standard Operating Protocols and verified to be noninfectious.

## Results

### The 1918 virus causes greater early tissue damage than VN/1203

We previously used a macaque infection model to examine the host response to the reconstructed 1918 virus at 3, 6, and 8 days after infection [8]. That study revealed that the 1918 virus caused a severe respiratory infection that culminated in acute respiratory failure and a fatal outcome. In addition, our histopathology and gene expression analyses suggested that events occurring well before the 3-day time point may have a critical influence on the course of disease progression. Furthermore, Baskin et al. [9] compared the host response to VN/1203 and A/Texas/36/91 containing two (HA and NA) or three (HA, NA and NS) genes from the 1918 pandemic virus in a macaque model at 1, 2, 4 and 7 days after infection. That study indicated that tissue damage induced by VN/1203 was the result of multiple factors such as an excessive and sustained type I interferon response and the deregulation of adaptive responses. In the present study, we sought to evaluate the host response at earlier time points after infection and to directly compare the host transcriptional response to these two highly pathogenic influenza viruses.

**Figure 1:**
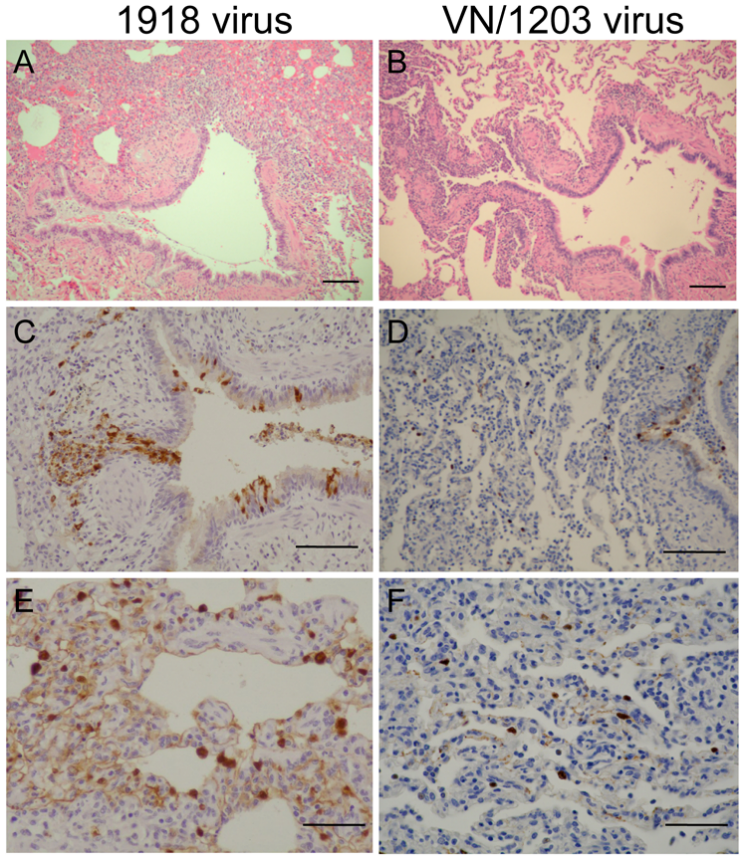
The 1918 virus causes greater early tissue damage than VN/1203 at 24 hours p.i. Panels A, C, E show 1918 infected monkey tissues at 24 hours p.i. Panels B, D, F show VN/1203 infected monkey tissues at 24 hours p.i. A. Peribronchiolar alveoli showed severe alveolitis, edema, and hemorrhage. Bar = 100 µm. B. Mild peribronchitis was observed, but alveoli were clear preserving air space. Bar = 100 µm. C. Large amounts of viral antigen were detected at bronchial epithelium in 1918 virus infected monkey lungs. Bar = 100 µm. D. Small amounts of viral antigens positive cells at bronchial epithelium in VN1203 virus infected monkey lungs. Bar = 100 µm. E. Large amounts of viral antigens at alveoli in 1918 virus infected monkey lungs. Bar = 50 µm. F. Scattered viral antigen positive cells at alveoli in VN1203 infected monkey lungs. Bar = 50 µm.

**Figure 2:**
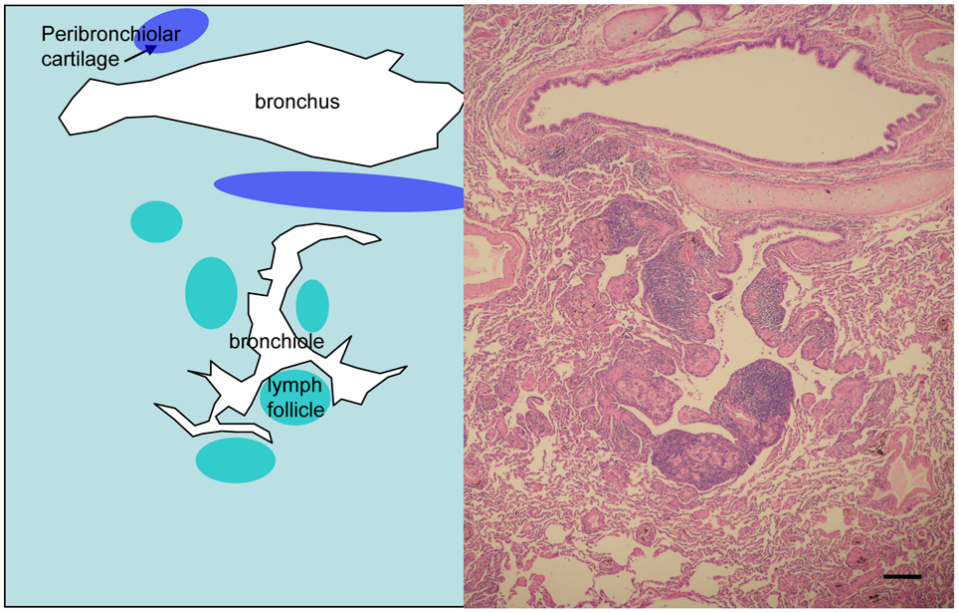
Macaques infected with the VN/1203 virus recovered from infection at 16 days p.i. Tissue regeneration and lymphocyte infiltration in mildly affected lung lobe containing prominent peribronchiolar lymph follicles development. Bar = 200 µm.

Animals infected with the 1918 virus or with VN/1203 were euthanized at 12, 24, or 48 h post-infection (p.i.). An additional group of animals infected with VN/1203 were euthanized at 3, 6, or 16 days p.i. Histopathology revealed that at 12 h p.i., the 1918 and VN/1203 viruses had already begun to cause pathology, and viral antigen was detected along the terminal and respiratory bronchiole (Figure S1). By 24 h p.i., antigen detection and severity of disease were more robust in the 1918-virus infected macaques, and the peribronchiolar alveoli showed severe alveolitis, edema, and hemorrhage (Figure 1, A). Additionally, the virus was widely distributed in bronchi and alveoli (Figure 1, C, E). This phenotype was lacking in macaques infected with VN/1203 (Figure 1, B, D, F). By 48 h p.i., the histopathology associated with 1918 and VN/1203 infection was similar, with clear alveolitis and alveolar edema around the bronchiole. Although the number of cells positive for viral antigen decreased in the edematous lesion, positive cells were still detected at the edematous lesion boundary (Figure S2). At later time points (days 3, 6, and 16), cells positive for viral antigen decreased in number in VN/1203-infected animals and by day 16 the lung showed prominent peribronchiolar lymph follicle development and tissue regeneration (Figure 2).

**Figure 3:**
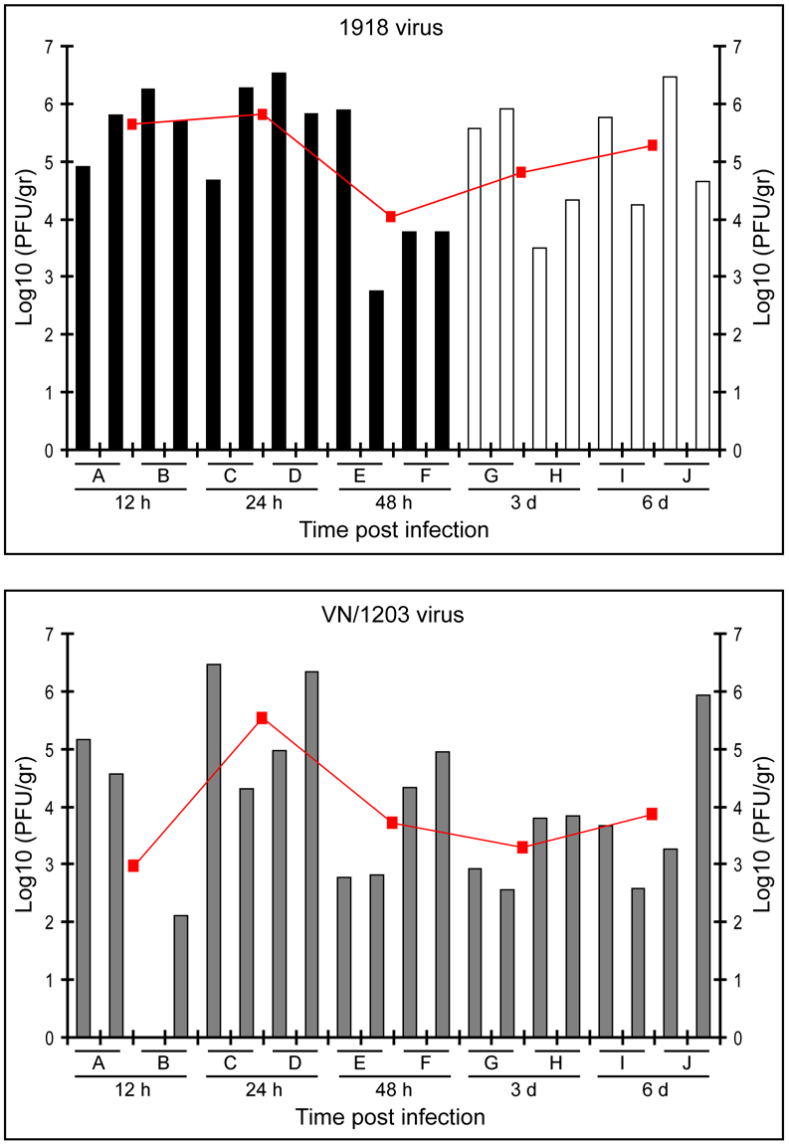
The 1918 virus overcomes the host response but not the VN/1203 virus. Virus titers were determined in plaque assays of bronchi homogenates at 12, 24 and, 48 h p.i. in animals infected with the 1918 and VN/1203 viruses, as well as 3 and 6 days p.i. in animals infected only with the VN/1203 virus. Each bar graph represents an individual measurement of viral load. Each individual animal (represented by the letters A to J) shows two measurements (left and right bronchi). Open bars in the 1918 virus titers graph at 3 and 6 days post infection were incorporated from Kobasa et al [8] for reference. Line graph shows the average of the four measurements per time point (two samples per animal, two animals per time point).

Analysis of viral titer revealed that at 12 h p.i., the titer of the 1918 virus appeared somewhat higher than the VN/1203 virus; whereas at 24 h p.i., titers for the two viruses were more similar (Figure 3). Remarkably, there was a decrease in viral titer for both viruses at the 48-h time point. Whereas we previously observed that the titer of the 1918 virus increased steadily at later time points (3, 6, and 8 days post infection) [8], VN/1203 titers did not increase after the 24-h time point and remained essentially constant at 3 and 6 days post infection (Figure 3). Thus, although 1918 and VN/1203 titers were similar at the 24-h time point, the 1918 virus had already caused greater pathology, which continued to worsen over time. In contrast, VN/1203 titers began to decrease after 24 h, and pathology began to diminish. These results suggest that macaques were able to mount a successful initial nonspecific innate response to both viruses as demonstrated by the similar decrease in titers during the 24–48 hour period. However, the 1918 virus appears to be able to overcome the host response and replicate at high levels [8], whereas VN/1203 replication was limited by the host response.

### The 1918 and VN/1203 viruses elicit similar interferon and cytokine transcriptional profiles

**Figure 4:**
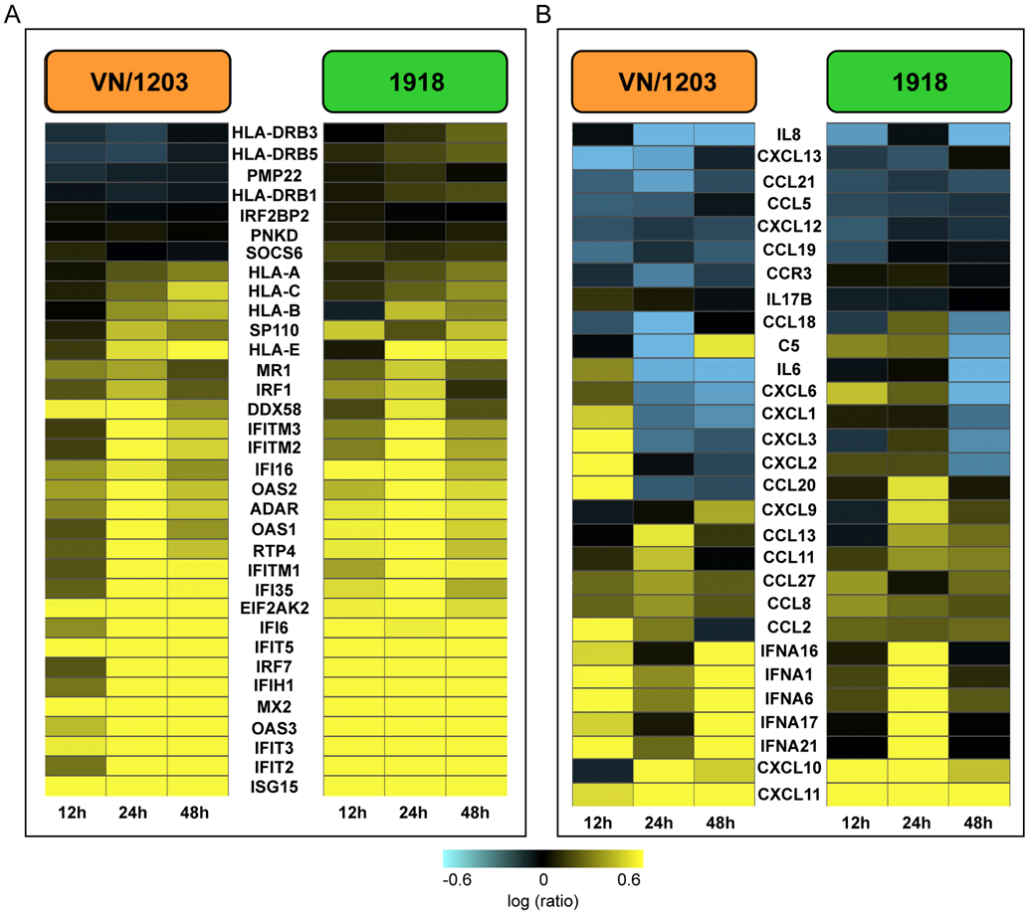
The 1918 and VN/1203 viruses elicit similar interferon and cytokine transcriptional profiles. Microarray analysis of 1918 and VN/1203 virus infected macaque bronchi at 12, 24 and 48 h p.i. A, Expression of type I interferon stimulated genes. B, expression of chemokines and cytokines related genes. Genes shown in yellow were up-regulated and those in blue were down-regulated in infected relative to mock-infected animals.

We used microarray analysis to determine whether the host transcriptional response to infection would provide insight into the differences in pathology and disease outcome observed for the 1918 and VN/1203 viruses. Our analyses revealed both similarities and differences in the transcriptional response to infection. For example, we found that the expression of type I interferon stimulated genes was up-regulated in response to both viruses as early as the 12-h time point, and the expression of these genes remained elevated at 24 and 48 h p.i. (Figure 4A). However, the 1918 virus elicited a stronger activation of these genes at 12 h p.i., but by 24 and 48 h p.i. this activation was higher with the VN/1203 virus (Table S1). ISG15 was one of the genes highly activated during infection with both viruses. It has been shown that ISG15 has antiviral activities against HIV-1 and Ebola [20],[21],[22], Sindbis [23],[24] and Influenza [25] viruses. Furthermore, ISG15 targets a wide variety of cellular pathways [26],[27],[28],[29],[30]. Consequently depending on the conjugation of ISG15, this could alter the host antiviral response or may impair viral replication.

We also analyzed the expression of selected chemokine and cytokine genes and again found a similar pattern of expression with a small transient difference at 24 h p.i. (Figure 4B). During our analysis we found that CXCL10 and CXCL11 were among the most highly induced chemokine genes during infection with both viruses. This illustrates a scenario where T cells are tethered to sites of inflammation at very early stages of infection [31]. A detailed listing of these genes and their change in expression can be found in Table S2. These results are consistent with our previous findings that both viruses elicit transcriptional induction of interferon- and cytokine-associated gene expression [8],[9]. However, even though the present direct comparison of the host response to these viruses indicates that the 1918 virus elicits a somewhat stronger induction of interferon-stimulated gene expression, it is unlikely that differences in this response alone would have a major impact on disease outcome.

### The 1918 and VN/1203 viruses differentially regulate the expression of inflammation and cell death related genes

**Figure 5:**
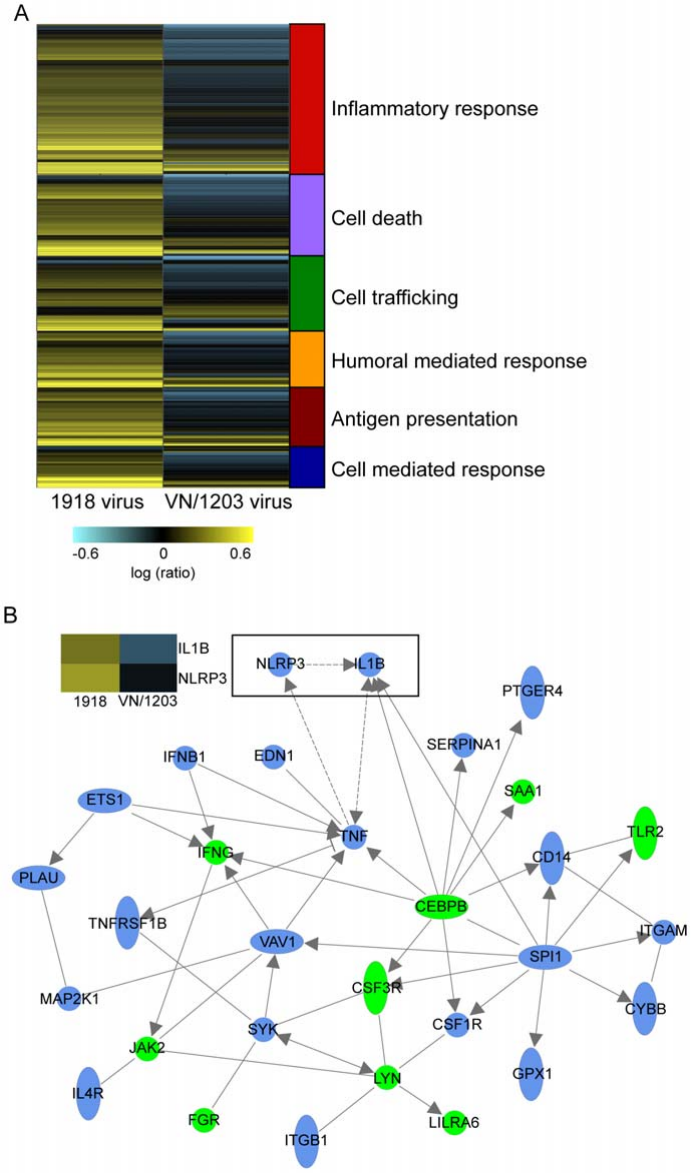
The 1918 and VN/1203 viruses differentially regulate the expression of inflammation and cell death related genes. (A) Venn diagram analysis was performed on the set of 428 genes that were differentially regulated by both viruses at 24 h p.i. All infected samples were compared to genotype-matched mock-infected samples via microarray analysis. Replicate samples were then pooled and error-weighted *in silico*. These genes were up-loaded into Ingenuity Pathway Analysis and categorized into functional categories that are included to the right of the heat map. (B) Biological network analysis of the top two functional categories: immune response (p-value = 2.42E-22) and inflammatory response (p-value = 1.16E-16) determined by Ingenuity Pathway Analysis. This analysis highlights a subset of genes that were up-regulated by both viruses (green shading) and a subset of genes that were anti-coregulated, that is up-regulated during 1918 infection but down-regulated during VN/1203 infection (blue shading). Boxed genes in the diagram highlight the anti-coregulation of the inflammasome components NLRP3 and IL-1β respectively. Included heat map illustrates the expression of IL1β and NLRP3. Yellow color indicates up regulation, blue indicates down regulation.

Our analyses also revealed groups of genes that were regulated differently by the two viruses. T-test analysis of individual time points indicated that there were 55, 428, and 68 genes that were differentially regulated by the two viruses at 12, 24, and 48 h p.i., respectively. Functional analysis of these genes indicated that the majority were grouped into the categories of inflammatory disease, immune response, and cell death (Table 1). Because the greatest differences in the host response occurred at 24 h p.i, we focused our analyses at this time point. Figure 5A shows a heat map of the 428 genes that were differentially regulated at 24 h p.i. Notably, many of these genes were up-regulated in the bronchus of 1918 virus infected animals but down-regulated in animals infected with VN/1203. This included numerous genes associated with the inflammatory response and cell death pathways. Expression of selected genes was verified by RT-PCR (Figure S4).

**Table 1:** Number of specific genes differentially regulated by 1918 virus infected bronchi relative to VN/1203 infected animals.

Function	12 h	24 h	48 h
Cell cycle	10	29	8
Cell death	22	107	19
Cellular growth and proliferation	18	113	20
Hematological disease	9	77	10
Immune response	6	107	13
Immunological disease	10	86	13
Infectious disease	1	39	3
Inflammatory disease	14	81	13
Respiratory disease	10	29	3

Because the largest number of gene expression differences elicited by the two viruses was associated with the inflammatory response, we used Ingenuity Pathways Analysis to evaluate this response in greater depth and to visualize the interconnections between individual genes. This software analyzes gene expression data in the context of known biological response and regulatory networks as well as other higher-order response pathways. A network of differentially expressed genes associated with the inflammatory response is shown in Figure 5B. In this Figure, genes depicted in blue were up-regulated by the 1918 virus but down-regulated by VN/1203 virus, whereas genes depicted in green were up-regulated by both viruses. Of particular note was the differential regulation of NLRP3 (nucleotide-binding domain and leucine-rich-repeat-containing protein 3) and IL-1β, key components of the inflammasome, a recently described component of the innate immune response to influenza A virus [32],[33],[34]. Verification of IL1β and NLRP3 expression are provided in Figure S5. By 24 h p.i. the 1918 virus elicited the up-regulation of these genes; however they were both down-regulated during VN/1203 infection. In addition, only the 1918 virus elicited the increased expression of TNF-α, a key mediator of the inflammatory response. This clear difference in the transcriptional regulation of this network of inflammatory genes may have a significant impact on eventual disease outcome.

### The 1918 and VN/1203 viruses elicit differences in the timing and extent of apoptosis after infection

**Figure 6:**
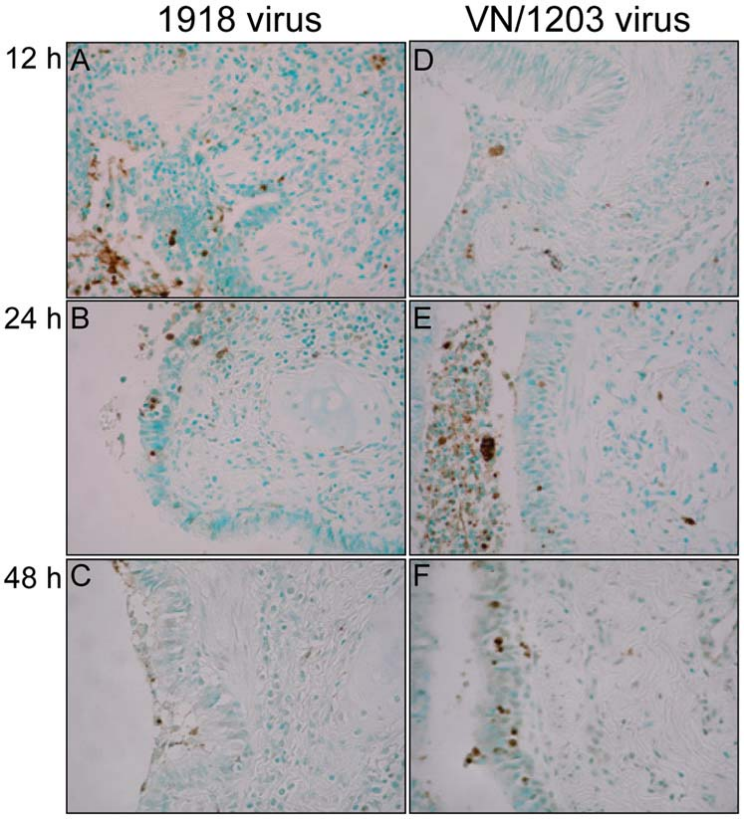
The 1918 and VN/1203 viruses elicit differences in the timing and extent of apoptosis after infection. TUNEL assay in fixed bronchi infected either with 1918 (panels A, B and C) or VN/1203 (panels D, E and F) viruses at 12, 24 and 48 h p.i. Positive cells (brown color) were desquamated and inflammatory cells. Additionally many phagocytes contained cells positive for apoptosis.

Our gene expression analyses also revealed that the 1918 and VN/1203 viruses perturbed the expression of genes associated with cell death, and that the two viruses elicited different patterns of gene expression associated with this response. We therefore sought to verify the presence of apoptotic cells by using the TUNEL assay in infected lung and bronchus. This assay revealed desquamated and apoptotic cells at the sites of inflammatory lesions as well as phagocytes containing cells undergoing apoptosis (Figure 6 and S3). In tissues from animals infected with the 1918 virus, there was evidence of apoptosis at 12 h p.i., but the level of TUNEL staining diminished markedly at later time points. In contrast, in tissues from animals infected with VN/1203, there was little evidence of apoptosis at 12 h p.i, but greater evidence of apoptosis was observed at the 24- and 48-h time points. These results indicate that both viruses elicited an apoptotic response in the lung and bronchus, but that the 1918 virus elicited an earlier response that later diminished, whereas VN/1203 elicited a comparatively delayed apoptotic response that was then maintained through the 48-h time point. Therefore, the timing and extent of the apoptotic response elicited by these two viruses may also have a significant impact on disease outcome.

## Discussion

The 1918 Spanish Flu pandemic was the deadliest infectious disease outbreak on record, and avian H5N1 influenza viruses have proven to be highly virulent in cases of human infection. To determine if there are differences in the host response to these two highly pathogenic influenza viruses, we performed extensive genomics analysis of bronchus tissue isolated from macaques infected with either the 1918 or VN/1203 viruses. We investigated the global transcriptional changes in bronchi, a lower respiratory tissue, rather than lungs because of the overwhelming cell infiltration that occurs in the lungs during influenza virus infection. Additionally, there was substantial replication of both viruses in this tissue. Our analyses revealed that these viruses elicited similar transcriptional profiles for interferon and cytokine genes, but markedly different transcriptional profiles for genes associated with inflammation and apoptosis. Our results depict a scenario where early during infection there was an accelerated up-regulation of the inflammasome by the 1918 virus in addition to the numerous cell death related genes that were up-regulated during infection with the 1918 virus but down-regulated during VN/1203 infection. These events represent important differences in the host response to these viruses and likely contribute to the severity and lethality of disease associated with 1918 virus infection.

Our previous study comparing macaque host responses to 1918 and A/Kawasaki/173/01 viruses showed that late after infection (3, 6 and 8 days) severity of tissue damage and fatal outcome was due to dysregulation of the antiviral response [8]. That study suggested that critical decisions influencing the outcome of the infection may occur very early. In this study, our results suggest that events taking place as early as 24 h after infection likely contribute to severity of tissue damage and lethal outcome during 1918 virus infection. For example, the 1918 virus elicited the increased expression of genes encoding NLRP3 and IL-1β, key components of the inflammasome, while these genes were being down-regulated by the VN/1203 virus. This finding suggests that an accelerated or excessive activation of the inflammasome is detrimental rather than protective in macaques. It is possible that early activation of NLRP3 and IL-1β production results in the infiltration of neutrophils and monocytes to the sites of infection where these cells continue to secrete cytokines resulting in a “cytokine storm”. Furthermore, Baskin et al. showed that VN/1203 infection of macaques was characterized by extensive tissue damage, an excessive and sustained type I interferon response, and innate immune induction [9]. Our present results also showed that VN/1203 caused significant pathology, but that 1918 infection generated more severe and sustained tissue damage. The study by Baskin et al. also compared the host response of VN/1203 infection to recombinant viruses containing two or three genes of the 1918 virus, whereas the present comparisons were performed using the fully reconstructed 1918 pandemic virus.

It is interesting to note that the virulence of these two viruses in macaques is somewhat different than in mouse models of infection. In the macaque model, the 1918 virus causes a lethal infection, whereas VN/1203 causes severe but typically non-lethal disease. In contrast, in the mouse model the avian VN/1203 virus is more pathogenic than the 1918 virus and is capable of causing disease and death at a much lower infectious dose. However, global transcriptional analysis of lung tissue from mice infected with the 1918 virus also revealed enhanced inflammatory and cell death responses that remained unabated until death [7]. Therefore, data from the mouse infection model also indicates a clear difference in the host response to 1918 and VN/1203 virus infection.

In addition to discovering significant differences in the inflammatory response elicited by the 1918 and VN/1203 viruses, we also identified notable differences in the apoptotic response induced by these viruses. Even though many studies have shown that influenza virus infection induces apoptosis, there are still contradicting reports about the induction and biological consequences of this response. For example, ectopic expression of the viral NS1 protein induces apoptosis [35]. In contrast, a NS1 deletion mutant has been shown to be a stronger inducer of apoptosis than the wild-type virus [36]. Regarding the consequences of the response, *in vitro* studies have demonstrated that expression of the anti-apoptotic protein Bcl2 results in impaired influenza virus production [37]. Caspase inhibitors also impair the propagation of influenza virus, possibly due to the nuclear retention of viral ribonucleoprotein (RNP) complexes [38]. Caspases may regulate the export of RNPs by increasing the diffusion limit of nuclear pores, thus allowing the passive diffusion of larger proteins [39]. Consequently, early during infection RNPs are transported out of the nucleus via an active export mechanism. As viral infection progresses and caspase activity increases cellular proteins are destroyed, thereby compromising the export of viral proteins. However, widening of nuclear pores may allow viral RNPs to use an alternative mode of exit from the nucleus thereby supporting viral replication. It has been suggested that this may allow influenza virus to take advantage of host cell antiviral responses to support viral replication [40].

Despite these apparent contradictions, differences in the activation of cell death pathways could help to explain the patterns of virus titers observed in our study. For example, the increase in apoptosis observed in the VN/1203 infected tissues (Figure 6, panels E, F) at the 48-h time point could result in less viral progeny as a consequence of cell death. In contrast, in the tissues infected with the 1918 virus, apoptosis decreased over time (Figure 6, panels A–C), which may ultimately provide a more favorable environment for the generation of additional viral progeny. The timing and extent of the apoptotic response elicited by these two viruses may therefore have a significant impact on eventual disease outcome.

We also found that a number of genes differentially regulated during 1918 virus infection were associated with cellular growth and proliferation and cell-cycle regulation (Table 1). The 1918 virus may take advantage of the altered expression of these host factors for utilization during its life cycle. *In vitro* experiments have demonstrated that nuclear extracts from uninfected cells increase influenza RNA synthesis as well as that of ectopically expressed virion RNPs, suggesting that cellular factors are involved in the switch between mRNA transcription and genome replication [41]. For example, RAF1 (RNA polymerase activating factor 1; identical to HSP90) appears to associate with the viral PB2 protein. This interaction may facilitate the association of unbound polymerase with RNA template, which could affect the interaction between PB1 and PB2 and therefore modulate the polymerase complex [42],[43],[44]. The involvement of host factors during the various stages of the viral life cycle is poorly understood and is the subject of major efforts to decipher the complex virus-host interactions that determine the outcome of infection.

In summary, our findings suggest that differential changes in the expression of inflammatory and cell death related genes as early as 12 to 24 h after infection likely contribute to the severity and lethal outcome of 1918 virus infection. Specifically, the accelerated up-regulation of the inflammasome components NLRP3 and IL-1β, as well as TNF-α, elicited by the 1918 virus may enhance neutrophil and monocyte infiltration and generate a strong antiviral response that will be detrimental rather than protective to the host. These findings may have therapeutic implications, suggesting that drugs that limit the inflammatory response may help to reduce the lung pathology associated with severe influenza virus infection.

The feasibility of such approach has been recently demonstrated by Aouadi et al. [45], who showed that silencing of the Map4k4 kinase in macrophages by oral delivery of β1,3-D-glucan-encapsulated siRNA particles protected mice from lipopolysaccharide-induced lethality by inhibiting TNF-α and IL-1β production. In addition, Marsolais et al. [46] showed that local administration of the sphingosine analog AAL-R to mouse airways significantly decreased the release of a variety of cytokines and chemokines known to contribute to the cytokine storm effect, resulting in less cytopathology of alveolar cells and inflammatory driven congestion of air spaces. Finally, Aldridge et al. [47] reported that decreased trafficking of a specific subset of dendritic cells by treatment with the peroxisome proliferator-activated receptor-agonist pioglitazone reduced morbidity and mortality associated with highly pathogenic influenza A virus infection. Thus, drugs that limit the early inflammatory response may be of particular benefit in treating infections caused by highly pathogenic influenza viruses.

## Supporting Information

Figure S1 Histopathology examination of bronchi and alveoli infected with recombinant 1918 and VN/1203 virus at 12 hours post infection. Panels A and B showing bronchial images. Bar = 200 µm. Panels C and D showing alveolar images. Bar = 50 µm. A, 1918 virus-infected bronchi showed viral antigen expression along the terminal bronchiole and respiratory bronchiole (arrows). B, VN1203 virus-infected bronchi showed viral antigen expression along the terminal bronchiole and respiratory bronchiole. C, positive cells were cuboidal cells lining the junction between the ciliated epithelia and alveolar cells. In addition, plump-shaped alveolar cells at the peribronchiolar alveolus were positive for antigens. D, positive cells were bronchiolar cells at the bronchiolar/alveolar junction, plump-shaped type II alveolar cells and linear-shaped type I alveolar cells at the peribronchiolar alveolus are indicated.
(4.89 MB TIF)

Figure S2 Histopathology examination of bronchi and alveoli infected with recombinant 1918 and VN/1203 virus at 48 hours post infection. Panels A and B showing bronchiolar images; C and D showing alveolar images. Bar = 50 µm. A, viral antigens were detected at the bronchiolar area and the peribronchiolar alveolus. B, viral antigens were detected at the bronchiolar area and the peribronchiolar alveolus. C, both plump and linear antigen-positive cells were detected. D, many linear-shaped, antigen-positive type I cells were detected in the edematous lesions.
(4.84 MB TIF)

Figure S3 The 1918 and VN/1203 viruses elicit differences in the timing and extent of apoptosis after infection of macaque lungs early during infection. TUNEL assay in fixed lungs infected either with 1918 (panels A, B and C) or VN/1203 (panels D, E and F) virus. Positive cells (brown color) were desquamated and inflammatory cells, additionally many phagocytes contained apoptosis positive cells.
(3.58 MB TIF)

Figure S4 Bar graph showing the expression of selected genes. Quantitative real-time PCR (Taqman) analysis of selected genes detected in macaque infected bronchi.
(0.48 MB TIF)

Figure S5 Bar graph showing the expression of NLRP3 and IL1β genes. Quantitative real-time PCR (Taqman) analysis of selected genes detected in macaque infected bronchi.
(0.43 MB TIF)

Table S1 Ingenuity Pathway Analysis: Interferon type I regulated genes changed≥2 fold compared to mock (p≤0.01)
(0.07 MB PDF)

Table S2 Ingenuity Pathway Analysis: Chemokine and cytokine related genes changed≥2 fold compared to mock (p≤0.01).
(0.07 MB PDF)
