# Supplementary figures and images for: Lethal Influenza Virus Infection in Macaques Is Associated with Early Dysregulation of Inflammatory Related Genes
Source: PLoS Pathog. 2009 Oct 2;5(10):e1000604. doi: 10.1371/journal.ppat.1000604 (PMC2745659; doi:10.1371/journal.ppat.1000604)

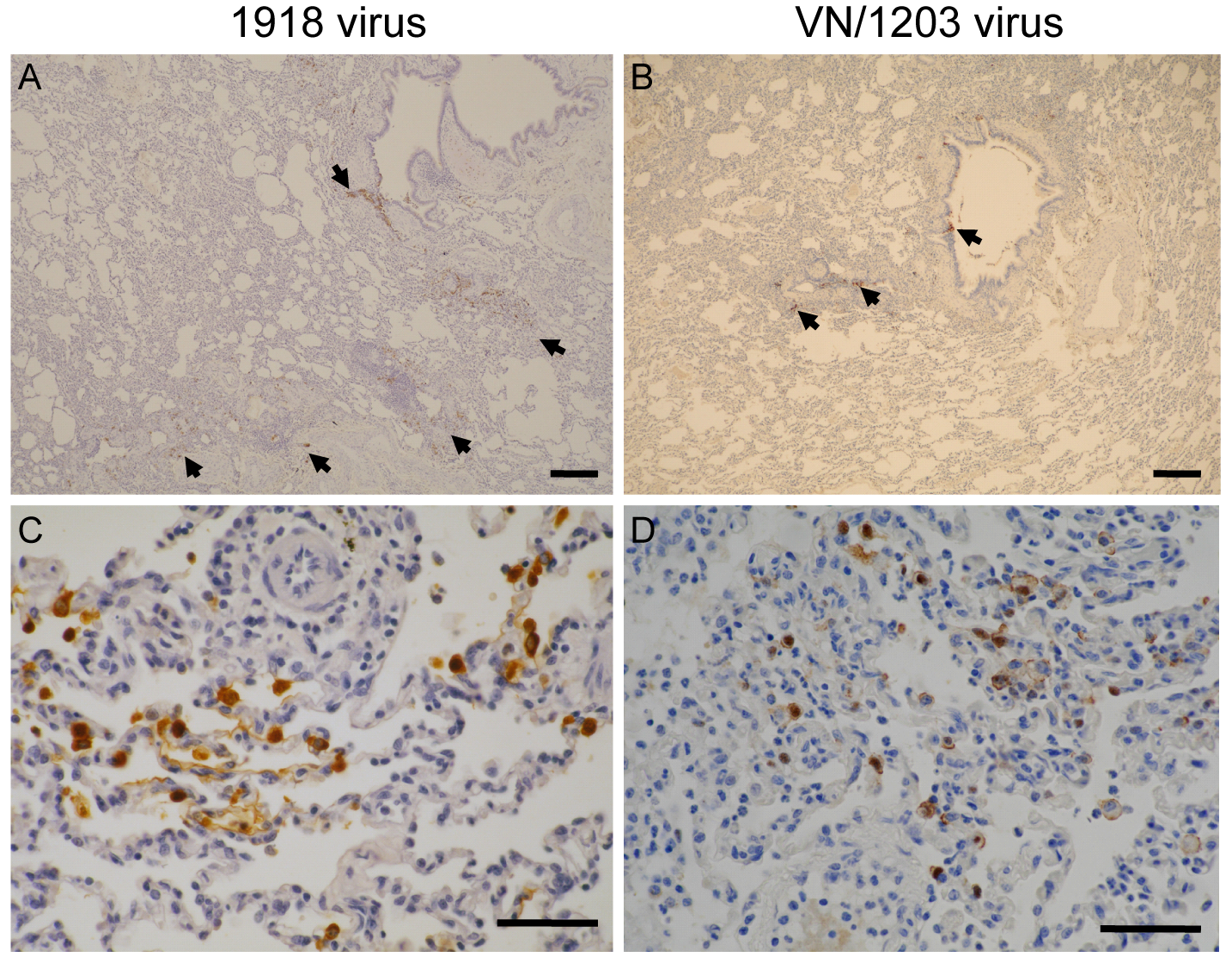

Supplement: Figure S1 — Histopathology examination of bronchi and alveoli infected with recombinant 1918 and VN/1203 virus at 12 hours post infection. Panels A and B showing bronchial images. Bar = 200 µm. Panels C and D showing alveolar images. Bar = 50 µm. A, 1918 virus-infected bronchi showed viral antigen expression along the terminal bronchiole and respiratory bronchiole (arrows). B, VN1203 virus-infected bronchi showed viral antigen expression along the terminal bronchiole and respiratory bronchiole. C, positive cells were cuboidal cells lining the junction between the ciliated epithelia and alveolar cells. In addition, plump-shaped alveolar cells at the peribronchiolar alveolus were positive for antigens. D, positive cells were bronchiolar cells at the bronchiolar/alveolar junction, plump-shaped type II alveolar cells and linear-shaped type I alveolar cells at the peribronchiolar alveolus are indicated. (4.89 MB TIF) [file ppat.1000604.s001.tif]

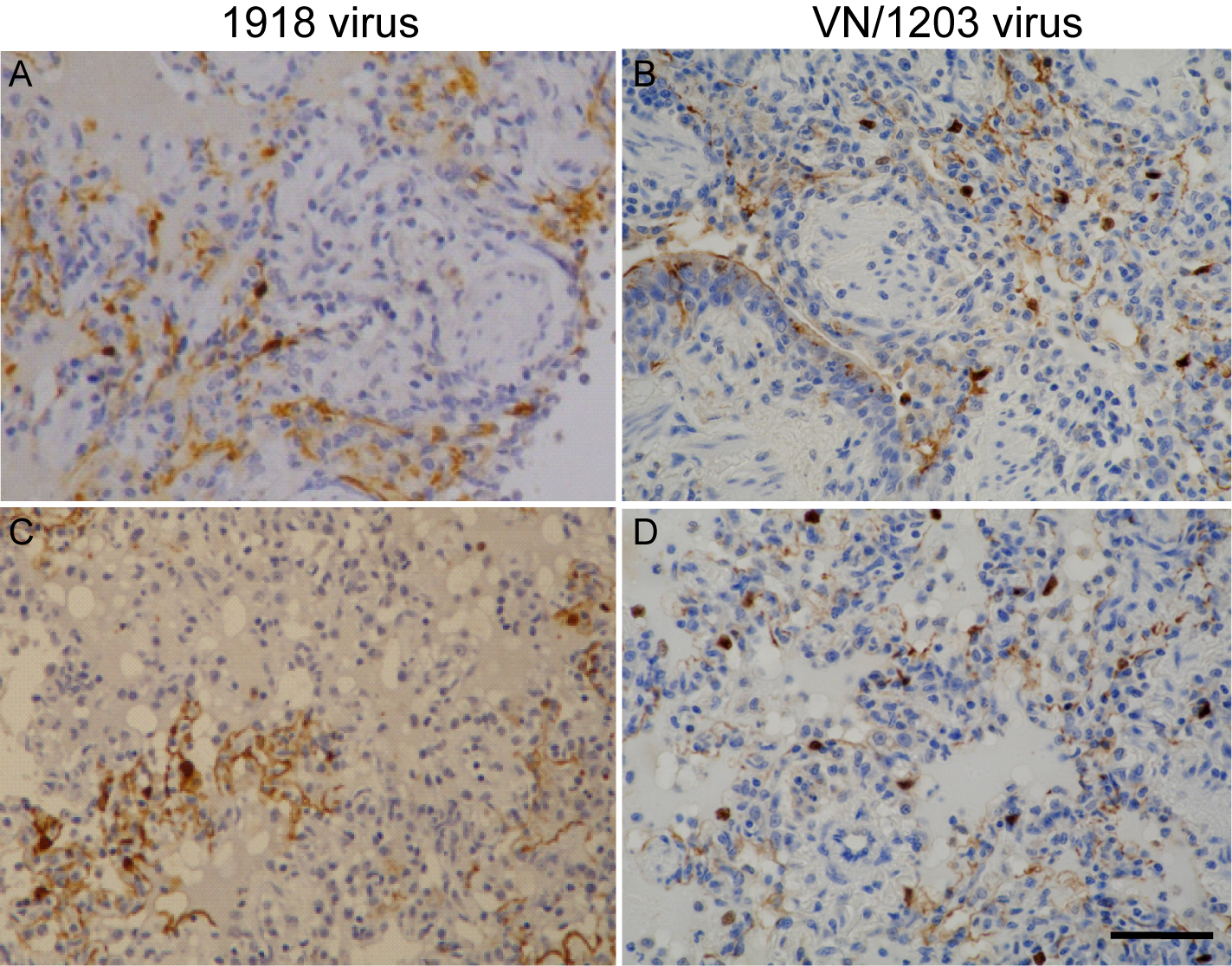

Supplement: Figure S2 — Histopathology examination of bronchi and alveoli infected with recombinant 1918 and VN/1203 virus at 48 hours post infection. Panels A and B showing bronchiolar images; C and D showing alveolar images. Bar = 50 µm. A, viral antigens were detected at the bronchiolar area and the peribronchiolar alveolus. B, viral antigens were detected at the bronchiolar area and the peribronchiolar alveolus. C, both plump and linear antigen-positive cells were detected. D, many linear-shaped, antigen-positive type I cells were detected in the edematous lesions. (4.84 MB TIF) [file ppat.1000604.s002.tif]

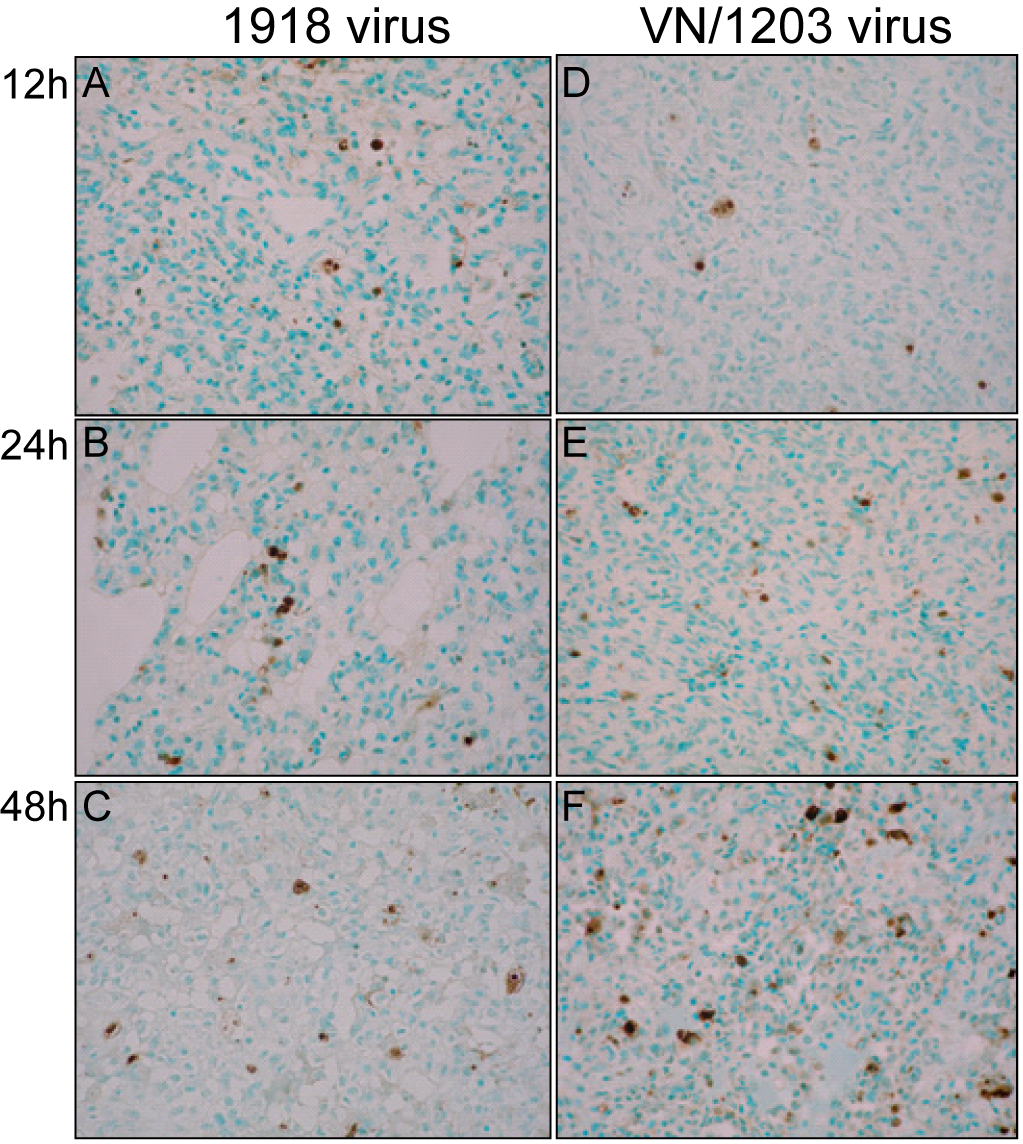

Supplement: Figure S3 — The 1918 and VN/1203 viruses elicit differences in the timing and extent of apoptosis after infection of macaque lungs early during infection. TUNEL assay in fixed lungs infected either with 1918 (panels A, B and C) or VN/1203 (panels D, E and F) virus. Positive cells (brown color) were desquamated and inflammatory cells, additionally many phagocytes contained apoptosis positive cells. (3.58 MB TIF) [file ppat.1000604.s003.tif]

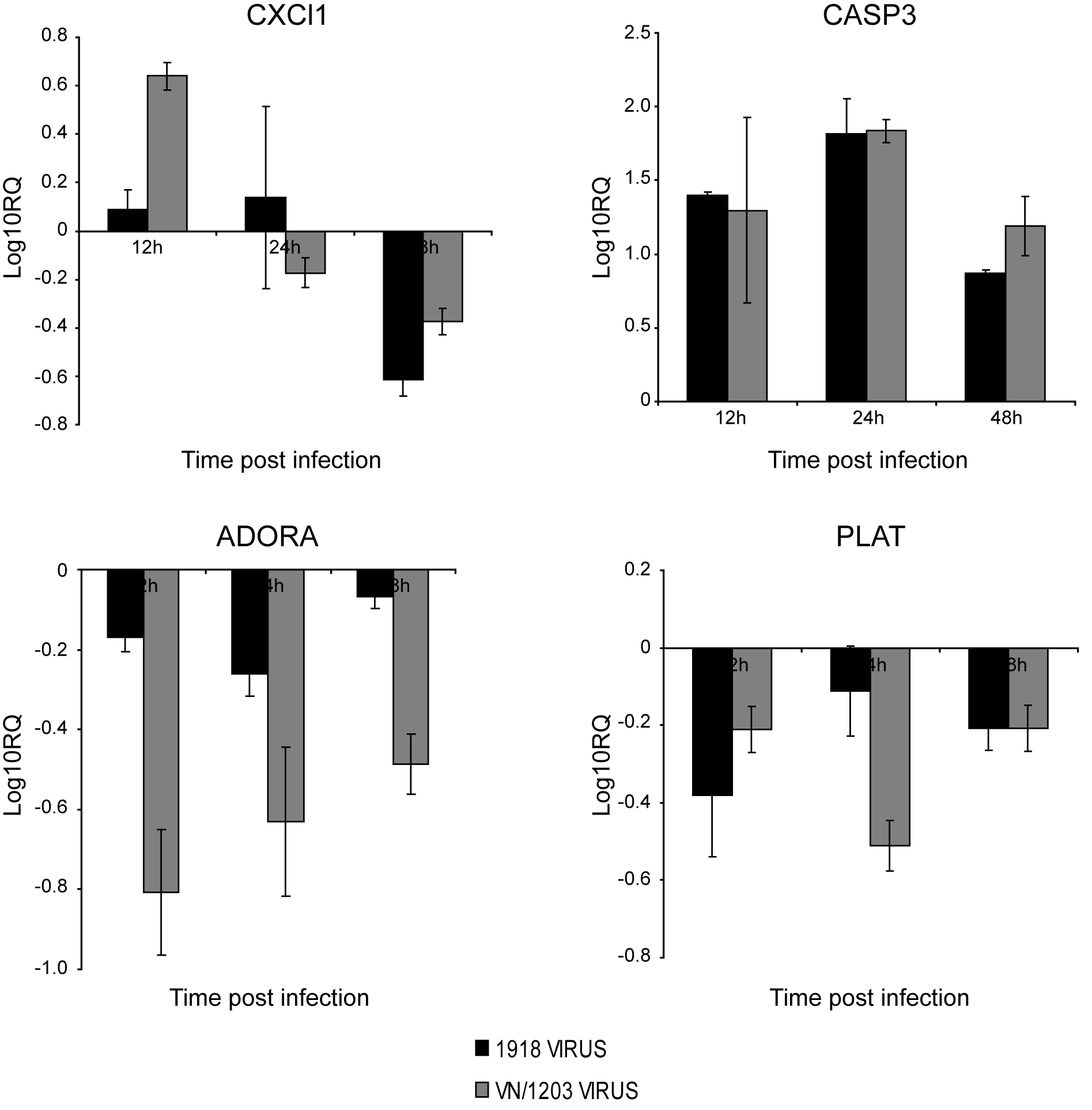

Supplement: Figure S4 — Bar graph showing the expression of selected genes. Quantitative real-time PCR (Taqman) analysis of selected genes detected in macaque infected bronchi. (0.48 MB TIF) [file ppat.1000604.s004.tif]

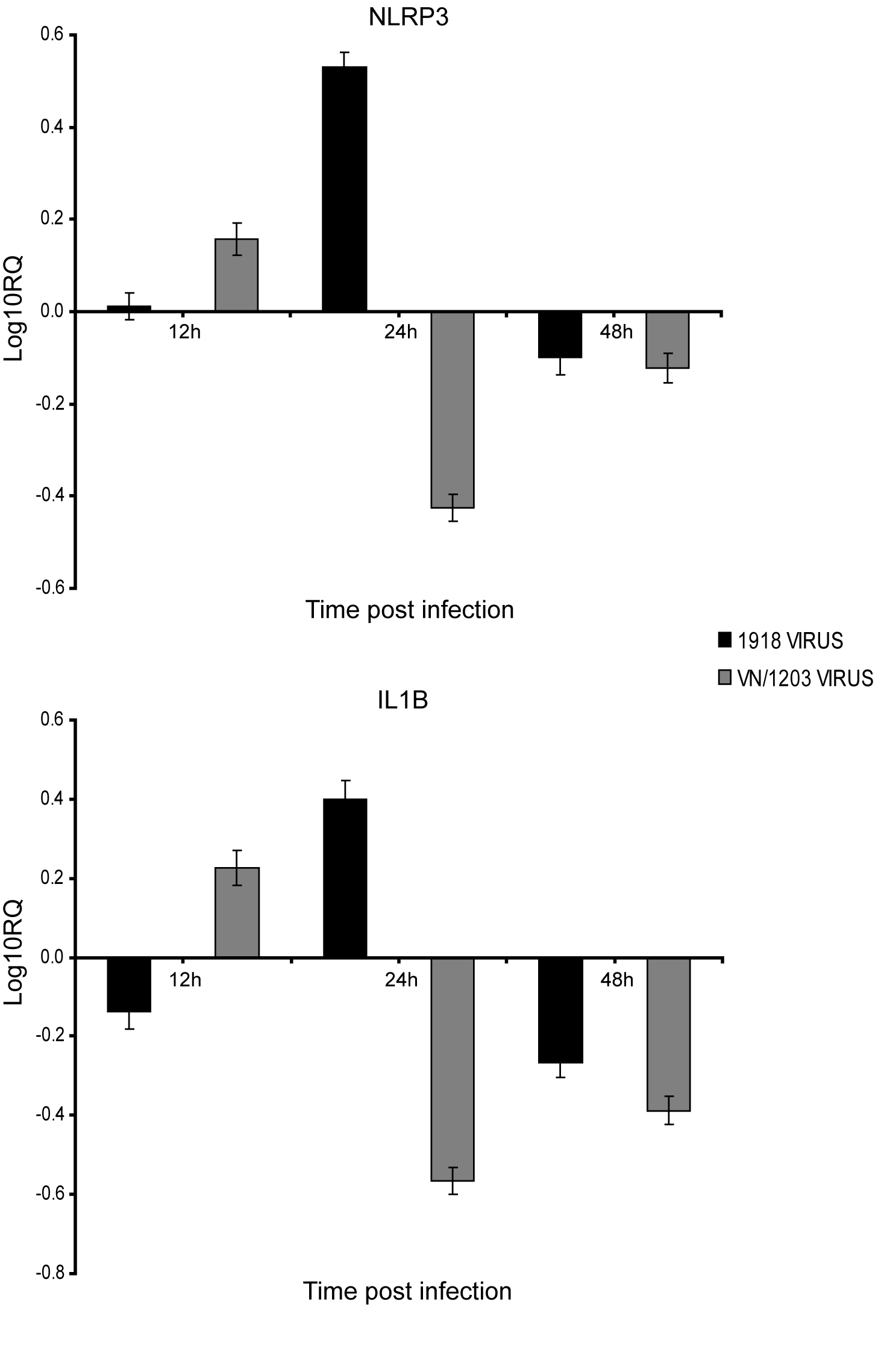

Supplement: Figure S5 — Bar graph showing the expression of NLRP3 and IL1β genes. Quantitative real-time PCR (Taqman) analysis of selected genes detected in macaque infected bronchi. (0.43 MB TIF) [file ppat.1000604.s005.tif]
