# Supplementary material for: Lethal Influenza Virus Infection in Macaques Is Associated with Early Dysregulation of Inflammatory Related Genes
Source: PLoS Pathog. 2009 Oct 2;5(10):e1000604. doi: 10.1371/journal.ppat.1000604 (PMC2745659; doi:10.1371/journal.ppat.1000604)
Supplement: Table S1 — Ingenuity Pathway Analysis: Interferon type I regulated genes changed≥2 fold compared to mock (p≤0.01) (0.07 MB PDF) [file ppat.1000604.s006.pdf]

Table S1. Ingenuity Pathway Analysis: Interferon type I regulated genes changed ≥ 2 fold compared to mock (p ≤ 0.01).

		VN/1203 virus						1918 virus					
		12 h		24 h		48 h		12 h		24 h		48 h	
Gene[(a)]	Entrez GeneID	FC[(b)]	PV	FC	PV	FC	PV	FC	PV	FC	PV	FC	PV
HLA-DRB3	3125	-1.442	0.008	-1.667	0.001	-1.125	0.636	1.007	0.967	1.305	0.212	1.730	0.000
HLA-DRB5	3127	-1.609	0.000	-1.708	0.000	-1.230	0.146	1.259	0.017	1.481	0.001	1.696	0.000
PMP22	5376	-1.433	0.124	-1.298	0.145	-1.234	0.032	1.145	0.088	1.311	0.056	1.067	0.333
HLA-DRB1	3123	-1.170	0.079	-1.318	0.027	-1.200	0.370	1.147	0.206	1.392	0.004	1.510	0.000
IRF2BP2	359948	1.091	0.537	-1.092	0.522	-1.039	0.793	1.140	0.188	-1.041	0.793	1.003	0.987
PNKD	25953	1.039	0.704	1.151	0.011	1.031	0.658	1.172	0.069	1.058	0.521	1.187	0.160
SOCS6	9306	1.232	0.209	-1.023	0.949	-1.124	0.703	1.460	0.000	1.274	0.192	1.382	0.029
HLA-A	3105	1.111	0.561	1.599	0.000	2.036	0.000	1.216	0.246	1.543	0.223	1.958	0.000
HLA-C	3107	1.198	0.538	1.820	0.000	3.197	0.000	1.315	0.002	1.690	0.025	2.195	0.000
HLA-B	3106	1.042	0.882	2.188	0.000	2.782	0.000	-1.281	0.043	2.794	0.000	2.092	0.000
SP110	3431	1.205	0.019	2.821	0.014	1.990	0.001	2.976	0.000	1.564	0.161	2.840	0.000
HLA-E	3133	1.374	0.318	3.321	0.000	4.317	0.000	1.156	0.209	4.084	0.000	3.554	0.000
MR1	3140	2.066	0.044	2.437	0.000	1.516	0.002	1.746	0.000	3.011	0.000	1.647	0.002
IRF1	3659	1.581	0.247	2.786	0.000	1.652	0.045	2.278	0.000	3.168	0.000	1.288	0.007
DDX58	23586	3.697	0.001	5.221	0.000	2.273	0.065	1.476	0.067	3.520	0.000	1.565	0.141
IFITM3	10410	1.405	0.484	5.299	0.000	3.118	0.000	2.058	0.000	4.908	0.000	2.419	0.000
IFITM2	10581	1.419	0.478	5.380	0.000	3.149	0.000	2.022	0.000	5.436	0.000	2.501	0.000
IFI16	3428	2.256	0.005	3.593	0.000	2.199	0.000	4.481	0.000	4.260	0.000	2.796	0.000
OAS2	4939	2.370	0.001	5.278	0.000	2.853	0.000	2.665	0.000	4.616	0.000	3.248	0.000
ADAR	103	2.096	0.062	4.758	0.000	3.042	0.000	3.480	0.000	5.307	0.000	3.518	0.000
OAS1	4938	1.548	0.000	7.269	0.001	2.234	0.092	3.674	0.023	4.845	0.006	3.126	0.016
RTP4	64108	1.659	0.218	12.079	0.000	2.854	0.000	3.549	0.000	5.405	0.000	2.840	0.000
IFITM1	8519	1.581	0.314	7.967	0.000	3.824	0.000	2.428	0.000	6.892	0.000	3.753	0.000
IFI35	3430	1.685	0.192	9.444	0.000	3.795	0.000	3.283	0.000	8.762	0.000	2.564	0.000
EIF2AK2	5610	3.847	0.005	8.053	0.000	4.425	0.000	3.669	0.000	4.458	0.000	3.307	0.000
IFI6	2537	2.160	0.014	6.825	0.000	6.247	0.000	4.411	0.000	3.639	0.000	6.193	0.000
IFIT5	24138	6.880	0.001	9.663	0.000	4.750	0.000	4.351	0.000	9.358	0.000	4.750	0.000
IRF7	3665	1.588	0.000	14.929	0.000	6.690	0.000	7.525	0.000	13.268	0.000	5.755	0.000
IFIH1	64135	1.898	0.299	15.349	0.000	4.885	0.000	9.124	0.000	11.134	0.000	7.668	0.000
MX2	4600	7.080	0.001	10.910	0.000	8.940	0.000	12.064	0.000	15.284	0.000	8.867	0.000
OAS3	4940	2.739	0.078	24.536	0.000	8.976	0.000	10.870	0.000	20.850	0.000	8.051	0.000
IFIT3	3437	3.589	0.000	26.280	0.000	15.388	0.000	16.959	0.000	23.511	0.000	14.672	0.000
IFIT2	3433	1.885	0.000	71.106	0.000	10.710	0.000	25.610	0.000	41.927	0.000	10.841	0.000
ISG15	9636	5.482	0.000	63.511	0.000	38.803	0.000	39.800	0.000	47.123	0.000	36.068	0.000

[(a)] Genes were selected by Ingenuity Pathway Analysis.

[(b)] Fold change (FC) and p-value (PV) calculations are detailed in Materials and Methods.
