# Supplementary material for: Lethal Influenza Virus Infection in Macaques Is Associated with Early Dysregulation of Inflammatory Related Genes
Source: PLoS Pathog. 2009 Oct 2;5(10):e1000604. doi: 10.1371/journal.ppat.1000604 (PMC2745659; doi:10.1371/journal.ppat.1000604)
Supplement: Table S2 — Ingenuity Pathway Analysis: Chemokine and cytokine related genes changed≥2 fold compared to mock (p≤0.01). (0.07 MB PDF) [file ppat.1000604.s007.pdf]

Table S2. Ingenuity Pathway Analysis: Chemokine and cytokine related genes changed ≥ 2 fold compared to mock (p ≤ 0.01).

		VN/1203 virus						1918 virus					
		12 h		24 h		48 h		12 h		24 h		48 h	
Gene[a]	Entrez GeneID	FC[b]	PV	FC	PV	FC	PV	FC	PV	FC	PV	FC	PV
IL8	3576	-1.113	0.681	-8.355	0.000	-12.586	0.000	-3.223	0.000	-1.142	0.709	-6.542	0.000
CXCL13	10563	-10.195	0.000	-3.490	0.000	-1.313	0.002	-1.572	0.006	-1.896	0.000	1.088	0.902
CCL21	6366	-2.093	0.000	-3.438	0.000	-1.790	0.003	-1.839	0.000	-1.539	0.003	-1.868	0.004
CCL5	6352	-2.094	0.000	-2.001	0.000	-1.195	0.351	-1.758	0.000	-1.625	0.001	-1.480	0.000
CXCL12	6387	-1.879	0.000	-1.544	0.000	-1.756	0.001	-2.012	0.000	-1.314	0.068	-1.484	0.000
CCL19	6363	-2.347	0.000	-1.445	0.000	-2.018	0.001	-1.853	0.000	-1.092	0.778	-1.170	0.668
CCR3	1232	-1.423	0.382	-2.665	0.014	-1.621	0.077	1.120	0.719	1.186	0.731	-1.105	0.693
IL17B	27190	1.344	0.059	1.153	0.071	-1.128	0.625	-1.271	0.062	-1.240	0.016	-1.020	0.860
CCL18	6362	-1.893	0.056	-7.119	0.000	1.011	0.972	-1.570	0.144	1.719	0.326	-2.797	0.000
C5	727	-1.092	0.835	-4.263	0.000	3.471	0.000	2.083	0.062	1.827	0.284	-3.530	0.000
IL6	3569	2.116	0.035	-3.803	0.000	-7.545	0.000	-1.156	0.110	1.072	0.944	-4.739	0.000
CXCL6	6372	1.620	0.014	-2.613	0.000	-3.406	0.000	2.860	0.000	1.685	0.083	-3.863	0.000
CXCL1	2919	3.023	0.000	-2.353	0.000	-2.974	0.000	1.196	0.122	1.173	0.775	-2.371	0.000
CXCL3	2921	4.364	0.000	-2.429	0.000	-1.940	0.000	-1.507	0.000	1.405	0.525	-2.926	0.000
CXCL2	2920	4.232	0.000	-1.127	0.409	-1.694	0.016	1.522	0.041	1.514	0.521	-2.712	0.000
CCL20	6364	4.105	0.000	-1.988	0.053	-1.780	0.036	1.208	0.392	3.402	0.106	1.147	0.728
CXCL9	4283	-1.206	0.523	1.081	0.800	2.509	0.004	-1.293	0.534	3.325	0.046	1.471	0.050
CCL13	6357	-1.025	0.958	3.458	0.000	1.348	0.043	-1.185	0.159	2.471	0.000	1.821	0.000
CCL11	6356	1.262	0.601	2.830	0.000	-1.047	0.639	1.404	0.062	2.238	0.000	2.017	0.000
CCL27	10850	1.783	0.003	2.341	0.023	1.659	0.219	2.280	0.002	1.125	0.803	1.788	0.141
CCL8	6355	1.728	0.151	2.243	0.000	1.597	0.000	2.211	0.003	1.767	0.000	1.563	0.000
CCL2	6347	9.389	0.000	1.955	0.000	-1.347	0.002	1.741	0.002	1.639	0.000	1.791	0.000
IFNA16	3449	3.175	0.040	1.123	0.763	8.268	0.000	1.181	0.489	4.387	0.168	-1.079	0.859
IFNA1	3439	3.863	0.002	2.137	0.117	7.523	0.000	1.461	0.468	4.201	0.099	1.269	0.650
IFNA6	3443	5.221	0.001	2.000	0.203	7.516	0.000	1.498	0.174	4.761	0.084	1.619	0.450
IFNA17	3451	3.119	0.015	1.140	0.830	7.621	0.000	1.053	0.949	38.513	0.000	1.020	0.969
IFNA21	3452	7.460	0.000	1.774	0.374	14.743	0.000	1.000	1.000	100.502	0.000	1.000	1.000
CXCL10	3627	-1.332	0.014	27.855	0.000	3.066	0.031	7.968	0.000	5.737	0.000	2.851	0.000
CXCL11	6373	3.279	0.301	15.703	0.000	7.084	0.001	5.769	0.000	6.004	0.000	3.876	0.000

[a] Genes were selected by Ingenuity Pathway Analysis.

[b] Fold change (FC) and p-value (PV) calculations are detailed in Materials and Methods.
